# R2R - software to speed the depiction of aesthetic consensus RNA secondary structures

**DOI:** 10.1186/1471-2105-12-3

**Published:** 2011-01-04

**Authors:** Zasha Weinberg, Ronald R Breaker

**Affiliations:** 1 Howard Hughes Medical Institute, Yale University, Box 208103, New Haven, CT 06520-8103, USA; 2 Department of Molecular, Cellular and Developmental Biology, Yale University, Box 208103, New Haven, CT 06520-8103, USA; 3 Department of Molecular Biophysics and Biochemistry, Yale University, Box 208103, New Haven, CT 06520-8103, USA

## Abstract

**Background:**

With continuing identification of novel structured noncoding RNAs, there is an increasing need to create schematic diagrams showing the consensus features of these molecules. RNA structural diagrams are typically made either with general-purpose drawing programs like Adobe Illustrator, or with automated or interactive programs specific to RNA. Unfortunately, the use of applications like Illustrator is extremely time consuming, while existing RNA-specific programs produce figures that are useful, but usually not of the same aesthetic quality as those produced at great cost in Illustrator. Additionally, most existing RNA-specific applications are designed for drawing single RNA molecules, not consensus diagrams.

**Results:**

We created R2R, a computer program that facilitates the generation of aesthetic and readable drawings of RNA consensus diagrams in a fraction of the time required with general-purpose drawing programs. Since the inference of a consensus RNA structure typically requires a multiple-sequence alignment, the R2R user annotates the alignment with commands directing the layout and annotation of the RNA. R2R creates SVG or PDF output that can be imported into Adobe Illustrator, Inkscape or CorelDRAW. R2R can be used to create consensus sequence and secondary structure models for novel RNA structures or to revise models when new representatives for known RNA classes become available. Although R2R does not currently have a graphical user interface, it has proven useful in our efforts to create 100 schematic models of distinct noncoding RNA classes.

**Conclusions:**

R2R makes it possible to obtain high-quality drawings of the consensus sequence and structural models of many diverse RNA structures with a more practical amount of effort. R2R software is available at http://breaker.research.yale.edu/R2R and as an Additional file.

## Background

Numerous structured RNAs have been identified in the last decade that are involved in a variety of biological processes [1-3]. Researchers are often aided by a graphical depiction of the consensus sequence and structural features of a given RNA class. Unfortunately, few tools have been designed to create such consensus diagrams [4,5], and available tools represent only sequence conservation or base-pairing probabilities in their output.

Several programs have been created to draw individual RNA molecules, and thus inherently address many of the issues associated with drawing a consensus diagram. Some programs implement algorithms that automatically determine a feasible layout of the RNA molecule [5-11], and several allow a user to adjust layouts interactively [8,12-14]. The layouts generated by these approaches are of practical value, and the automated approaches require minimal human effort. However, the resulting drawings are often not as readable or as aesthetic as those generated manually. To prepare high-quality diagrams for publications, researchers often use general-purpose drawing programs such as Adobe Illustrator. However, this approach is very time consuming.

The goal of R2R is to facilitate the creation of RNA consensus diagrams by bioinformaticians that are comparable in quality to those produced using Adobe Illustrator, but take less time to create. Because consensus diagrams are generally derived from multiple-sequence alignments, R2R takes as input a multiple-sequence alignment in Stockholm format [15] with added annotation to direct the drawing. R2R can also create depictions of single RNA sequences. Because of the effort still required to draw highly aesthetic diagrams, R2R is not usually appropriate for drawing raw candidate RNAs predicted by bioinformatics, but rather is intended primarily for use in preparing publications of RNAs. Using R2R, we have created over 100 RNA drawings in previous publications [16-19].

R2R source code is freely available and is distributed with over 100 example input and output files in both PDF and SVG format (Additional file 1). A user manual is also available (Additional file 2).

## Results

### An example consensus diagram

**Figure 1:**
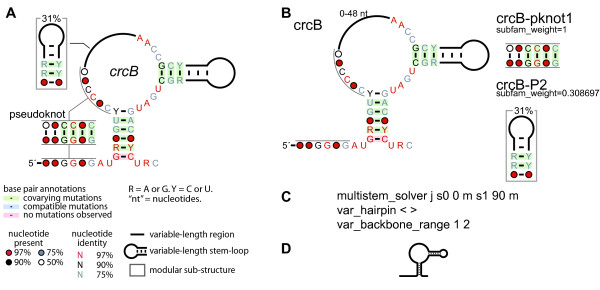
**Example of a consensus diagram for a noncoding RNA**. (A) Completed diagram of the consensus for the *crcB *motif [19] created using R2R and Adobe Illustrator. The consensus diagram shown here is modelled on a previously published figure [19]. The legend inset also applies to other consensus diagrams in this report. A generic legend is available for R2R users (Additional files 3 and 4). (B) Raw output generated by R2R when run on the *crcB *structure. The pseudoknot is depicted separately, along with the hairpin that is present in 31% of *crcB *RNAs. (C) R2R commands (Additional file 2) used for the main structure in part B. The symbols j, <, >, 1 and 2 in these commands refer to columns in the alignment (explained in Figure 2). (D) Raw output of R2R "skeleton" drawing of the *crcB *motif.

**Figure 2:**
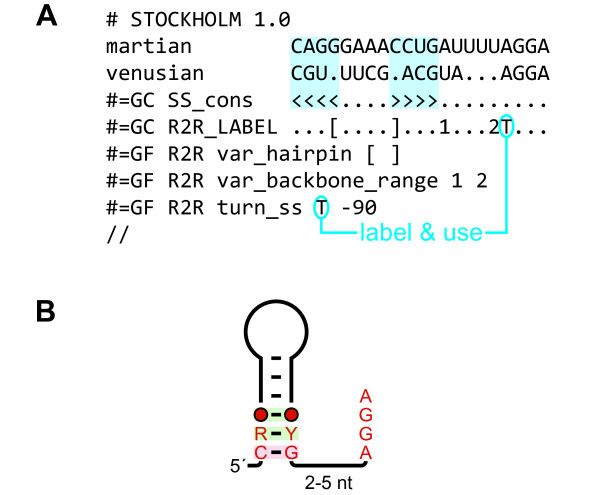
**Complete example using a tiny, contrived RNA**. (A) Alignment of fictional RNAs, in Stockholm format [15]. The "#=GC SS_cons" line specifies a stem (shaded blue rectangles) based on matching angle brackets (< and >). The "#=GC R2R_LABEL" line associates the labels [, ], 1, 2 and T with specific columns. The labels are used in R2R markup (e.g., see text "label & use"). (B) Raw output of R2R when run on the input in part A.

The *crcB *motif [19] was used to provide an example of a consensus diagram drawn using R2R (Figure 1A). The consensus is a representation of conserved sequence and secondary-structure features, the degree of conservation of nucleotides and a summary of covarying positions that retain base-pair complementarity. The output of R2R (Figure 1B) was customized by using additional commands (Figure 1C), and assembled using Adobe Illustrator into a finished diagram. Generic symbols and graphics used in finished diagrams are provided (Additional files 3 and 4). A complete example of R2R input and output is also given for a contrived RNA class with two representatives (Figure 2).

### Multistem junctions

**Figure 3:**
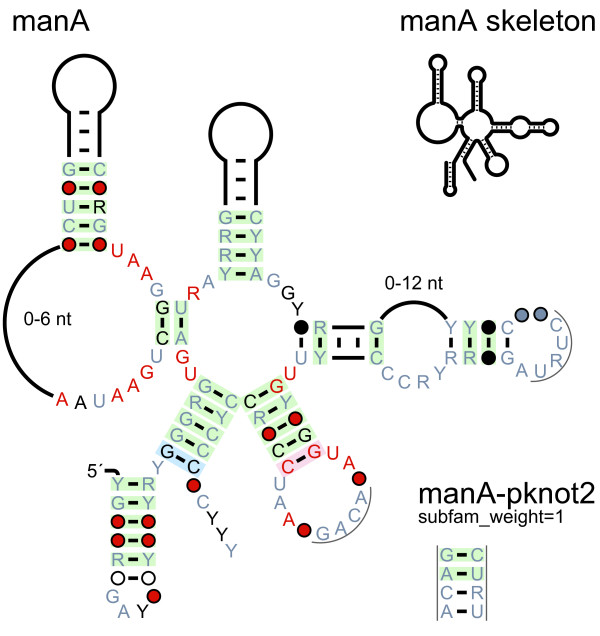
**Automated layout of multistem junctions**. Raw R2R output is shown for the *manA *motif [19], a pseudoknot (lower right) and a "skeleton"-style drawing (upper right). The layout of the central multistem junction was determined using R2R's solver functions. This motif presents an atypical case where it is not possible to direct all stems horizontally or vertically. The directions of the stems were chosen manually to promote symmetry, the lowest two stems on the junction were constrained to be aligned vertically, and the midpoint of these two stems was aligned horizontally with the upper-most stem. Subject to these constraints, R2R's solver chose a layout that best approximated a circle. The layout was used in a previously published figure [19]. A finished drawing would require assembling the pseudoknot (as in Figure 1), and moving text. Modular structures present in some *manA *RNAs are not shown. Note: the text "0-6 nt" was moved inward manually to fit the column width.

Nucleotides within multistem junctions and internal loops are typically positioned along a circle (e.g., as in Figure 1). Like most RNA-drawing programs, R2R supports manual layout of such loops, as well as a circular layout in which stems are oriented in whatever directions fit the circle. R2R also supports the drawing of loops that approximately follow a circle, subject to constraints on the directions of their stems (Figure 3). These constraints are specified by the user, and can be used to avoid overlapping nucleotides elsewhere in the diagram, to orient all stems in horizontal or vertical directions, or otherwise to promote symmetry in stem directions. Stems within the multistem junction can also be constrained to align horizontally, vertically or in an arbitrary axis. The resulting problem is expressed as a non-linear program (see Implementation), and solved by CFSQP [20]. This feature accelerates the determination of an approximately circular layout, compared to manual trial and error.

### Pseudoknot drawing styles

**Figure 4:**
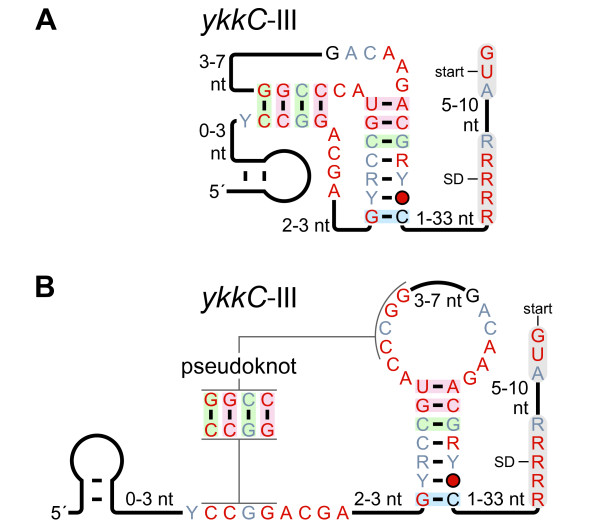
**Pseudoknot depiction styles**. The *ykkC*-III motif [19] is used to illustrate two styles of drawing pseudoknots (A) "In-line" style. (B) "Callout" style. A portion of this figure was adapted from a previous report [19].

R2R supports two styles to show pseudoknots. In an "in-line" style, pseudoknot pairings are drawn directly (Figure 4A). The pairing relationships are often most clear in the in-line style, but this layout is not possible for many RNA secondary structures without making other compromises. By contrast, the "callout" style (Figure 4B) involves connecting distant base-paired regions with a line marked "pseudoknot". The pseudoknot pairings can be shown explicitly in a small callout drawing. The callout allows annotation of covariation data, and helps the reader to see precisely which nucleotides form base pairs.

### Modular structures

**Figure 5:**
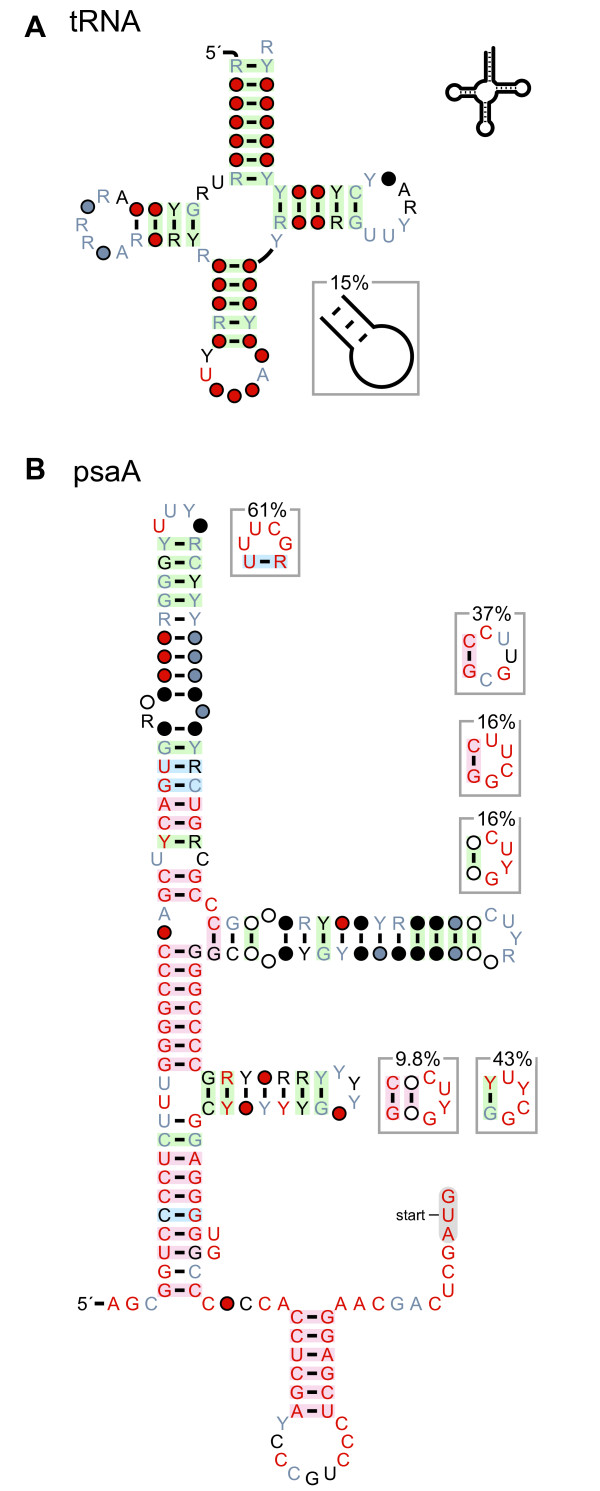
**Modular structures in tRNAs and *psaA *RNAs**. (A) Consensus diagram of tRNAs taken from the Rfam database [29] and drawn using the standard tRNA layout. A hairpin that is only sometimes present is shown (lower, right). In this case, the hairpin does not conserve obvious features, and is therefore shown in the "skeleton" style. Other consensus features of tRNAs are not depicted here. (B) Consensus diagram of *psaA *RNAs [19]. The terminal loops of this RNA often adopt the UNCG tetraloop [21], but also often conform to the CUNG tetraloop [30] or an unstudied CYYGN pentaloop pattern. These distinct sequence features are drawn as modular structures, and were manually positioned near to their associated terminal loop. Other than this repositioning, the diagram is raw R2R output. Some additional annotation and sequence of the *psaA *motif is not shown here. A portion of this figure was adapted from a previous report [19].

Many RNA motifs exhibit modular sub-structures that are present in only some motif representatives. For example, in many RNA motifs, certain hairpins are absent in some representatives, and some terminal loops frequently adopt one or more well-defined sequences (e.g., either GNRA or UNCG [21]). To show a modular structure, the R2R user uses regular expressions or Boolean logic to define which motif representatives exhibit the modular structure (Additional file 2). The occurrence frequency of the modular structure is automatically calculated by R2R (Figures 1 and 5).

### Drawing of individual RNA molecules

**Figure 6:**
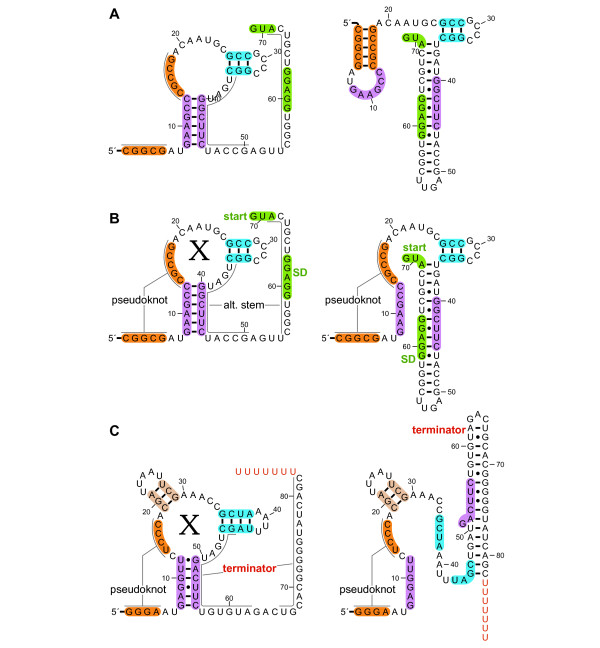
**Alternate structures of *crcB *RNAs**. (A) Output of R2R for predicted alternate structures for a *crcB *RNA in *Acidothermus cellulolyticus *11B. Stems are shaded so that their positions in the alternate structures are apparent. R2R commands were used to shade selected nucleotides, to position the multistem junction using the automated solver and to turn the direction of the backbone in two places within the 3' tail. (B) Finished drawing, assembled using Adobe Illustrator based on part A. The predicted Shine-Dalgarno (SD) sequence and start codon are shaded green and labelled. It is hypothesized that when the RNA binds its ligand "X" (left), the SD sequence is available for ribosome binding, allowing gene expression. In the absence of ligand (right), the SD sequence is sequestered, inhibiting gene expression. This latter drawing was made by combining the two drawings in part A. (C) Alternate hypothetical structures of a *crcB *RNA in *Roseburia intestinalis *L1-82, finished drawing. The hypothesized structure without the ligand X (right) allows the formation of a putative transcription terminator, which inhibits gene expression. The terminator stem is labelled, and its characteristic poly-U stretch is colored red.

Although the primary goal during the design of R2R was to produce software to assist in drawing consensus diagrams, R2R can also be used to draw the sequences and structures of individual representatives of a noncoding RNA class. For example, Figure 6 shows alternate structures possible in *crcB *RNAs from *Acidothermus cellulolyticus *and *Roseburia intestinalis *that suggest a model for gene regulation. We also previously used R2R to display structural probing data obtained by in-line probing experiments on a SAM-IV riboswitch [17].

### Design principles for RNA secondary structural diagrams

R2R facilitates the application of the following design principles for RNA secondary structure diagrams. Although little research has investigated practical benefits of different RNA drawing styles [10], the principles integrated into R2R are similar to broadly followed guidelines for RNA depictions [10,22] and some are related to ideas accepted in the field of graph layout [10,23].

The principles are as follows. First, nucleotides or other symbols should not overlap. Second, nucleotides within bulges and loops should ideally be drawn along circles. Such a layout leads to symmetry [23] in the looped nucleotides, which share a common property. The circular layout also avoids arbitrarily drawing attention to individual nucleotides that might otherwise be located on a corner. Third, stems should ideally run horizontally or vertically, to emphasize the common structural role of stems. Fourth, the distance between consecutive nucleotide positions along the RNA backbone should be constant throughout the diagram. This principle avoids inelegant bunching of nucleotides, or extra space between nucleotides that draws unwarranted attention or requires additional clarification for the user to follow the RNA backbone. Finally, the diagram should be compact, which is both aesthetic and space-saving. Some of these principles often conflict, and the inference of an optimal solution may require some manual intervention.

Consensus diagrams merit annotation to highlight the extent of nucleotide conservation and to feature evidence supporting the proposed structure. This information, which is included in Figure 1, is automatically calculated by R2R (see below). Other annotations useful in consensus diagrams are the depiction of variable-length, poorly conserved regions as well as modular structures. R2R supports such annotations (e.g., see Figure 1), based on the user's explicit judgment regarding the RNA motif data.

### Automatically calculated consensus annotation

Some annotations specific to consensus diagrams are automatically computed by R2R, using approaches described previously [16]. R2R graphically depicts the extent of conservation at nucleotide positions within an RNA. To reduce bias caused by highly similar sequences, sequences are weighted by the GSC algorithm [24] as implemented by the Infernal software package [25]. If the weighted frequency of a nucleotide exceeds 75%, R2R draws the nucleotide with a specific color (e.g., Figure 1) to indicate whether its frequency exceeds 75%, 90% or 97%, although these parameters can be adjusted. Otherwise, if the nucleotide is a purine or pyrimidine with a frequency above 75%, R2R indicates whether this frequency exceeds the same thresholds. The conservation of purine or pyrimidine identity is often associated with structural constraints. If a position does not meet the preceding criteria, R2R reports whether a nucleotide is present in the position with weighted frequency more than 50%, 75%, 90% or 97%, or otherwise does not show the nucleotide position.

R2R does not indicate other patterns of conservation. For example, the nucleotide immediately 5' to the hammerhead ribozyme cleavage site must be A, C or U [26], but this will not be indicated automatically by R2R. However, we concluded that routinely annotating such conservation patterns would unduly complicate diagrams, and users could add such distinctions that are desired. We also considered using entropy [27] as a measure of conservation. Although entropy measures conservation in a more general manner, we found it difficult to develop an intuition for how specific levels of entropy relate to likely biochemical constraints.

R2R marks each predicted base pair to indicate covariation (e.g., Figure 1). If two RNAs can form a Watson-Crick or G-U base pair at equivalent locations, and the base pair identities differ at both positions (e.g., A-U in one sequence and C-G in another), R2R classifies the base pair as covarying. If they vary at only one position (e.g., A-U in one sequence and G-U in another), the base pair is considered to carry a compatible mutation. Base pairs whose nucleotides are invariant have no mutational evidence for or against such base-pair predictions, and are marked accordingly. Each of these classifications is indicated unobtrusively by shading the base pairs with specific colors. Positions that contain non-canonical base pairs with a frequency exceeding 10% are not shaded.

This automated R2R annotation does not reflect the extent or confidence of covariation. While such information can be useful, we believe that thorough evaluation of covariation evidence ultimately requires analysis of the full sequence alignment. For example, misleading covariation can result from an incorrect alignment of sequences, or from alignments of sequences that do not function as structured RNAs. Unfortunately, there is no accepted method to assign confidence that entirely eliminates the need to analyze the full alignment.

### User effort required with R2R

Since R2R's overriding goal is to facilitate highly aesthetic diagrams, it requires the user to give it explicit instructions to customize the RNA layout (e.g., Figure 1C), and to edit R2R's raw output in a general-purpose drawing program (e.g., compare raw Figure 1B with finished Figure 1A). In our experience, this manual effort is usually modest. The ~800-nucleotide GOLLD RNA [18] structure took us roughly 16 hours to draw using R2R, mainly owing to the challenge of finding a layout that fits within a page. However, most RNAs are hairpin structures that do not require any kind of customization, and were easily drawn in minutes. RNAs with complex structural features (e.g., pseudoknots or multistem junctions where the default layout is unsatisfactory) or annotations (e.g., modular structures or nucleotide positions with special significance) were still usually completed within 30-60 minutes.

### Limitations

Despite the capabilities offered by R2R, we see some areas for improvement. First, a graphical user interface would allow additional researchers to more easily use R2R, and could help to make some tasks even faster for all users. Second, numerous features are possible to enrich diagrams with additional layout, particularly for RNAs with unusual biochemical features. Third, further automation of layout selection would speed the use of R2R. Fourth, R2R is also not designed to implement schematic diagrams that display extensive tertiary interactions or to project diagrams that are positioned to better reflect positions of nucleotides or substructures in atomic-resolution models (e.g., the newer secondary structure format for group I introns [28]).

## Implementation

### Default layout of loops

By default, all nucleotides in a loop are positioned along a common circle. R2R keeps the distance between consecutive nucleotides strictly constant. Previous solutions to this problem assumed that distance between base-paired nucleotides is equal to the distance between consecutive nucleotides [22], but these distances are not assumed to be equal in R2R. Given a radius *r*, the angle between nucleotides along the circle is calculated based on the isosceles triangle with sides *r, r *and *d*, where *d *is the fixed distance between consecutive nucleotides or between base-paired nucleotides. Suppose r* is the radius of a suitable circle for a given loop. If nucleotides are drawn with radius *r *<r*, the sum of angles will exceed 360 degrees, while *r *>r* will result in fewer than 360 degrees. R2R uses binary search to solve for r*. A similar approach was also developed for VARNA [13]. For bulges or a side of an internal loop, the angles should sum to 180 degrees.

### Layout of multistem junctions expressed as a non-linear program

In many RNA structures, it is desirable to arrange the nucleotides within a multistem junction on a circle, while constraining the stems on the junction to be oriented in specific directions (e.g., Figures 1 or 3). These directions are typically dictated by a desire to avoid overlaps in other parts of the diagram, or to promote consistency or symmetry in overall stem directions. The stem-direction constraints make it impossible in general to follow a perfect circle, but a close approximation is usually feasible. In R2R, the stem directions are specified by the user, which avoids creating a much harder global optimization problem for the computer.

This layout problem is formulated in R2R in terms of a non-linear program (NLP), consisting of a non-linear objective function that is minimized subject to non-linear constraints. The NLP is solved by CFSQP [20]. Gradients of objective or constraint functions, which are used by CFSQP, are calculated using automatic differentiation. In automatic differentiation, mathematical functions are built using an abstract numeric data type that represents a symbolic expression, and the resulting symbolic expression is differentiated recursively.

R2R provides three different mathematical formulations to express the notion that the junction should approximate a circle. All methods use highly non-linear functions, and the optimizer can get stuck in local minima. Therefore, it is sometimes useful to try multiple formulations, although the second formulation usually produces an acceptable layout (Additional file 2).

**Figure 7:**
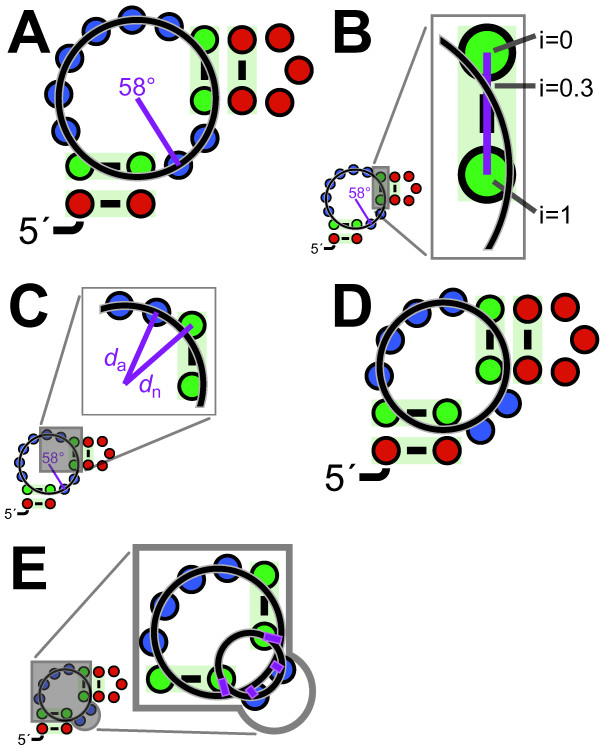
**Multistem-junction layout as a non-linear program**. (A) Illustration of first formulation, with circle drawn. The unpaired nucleotides on the junction (blue circles) perfectly fit the circle by construction, while the paired nucleotides (green circles) do not. The purple line indicates the angle of the nucleotide 5' to the enclosing pair, whose optimal value is roughly 58°. (B) The line connecting two paired nucleotides is shown in purple, and different intersection points are shown, from 5' nucleotide (i = 0) to 3' (i = 1). The optimal value is roughly i = 0.3. (C) Two purple lines mark the distance *d*_n_ from the circle's center to a base-paired nucleotide, and *d*_a_ from the circle's center to the adjacent nucleotide. Each of the four base-paired nucleotides in this example will contribute a term (*d*_n_-*d*_a_)^2 ^to the objective function. (D) Illustration of second formulation, using an example chosen so that some nucleotides would deviate significantly from the main circle. This circle is again shown explicitly. (E) The lower right unpaired region deviates from the main circle, but is positioned along an independent circle, which is shown. The four purple lines indicate deviations from this independent circle to the target main circle. Each of these lines corresponds to a term in the objective function.

In the first formulation, unpaired nucleotides in the junction are forced to lie on a common circle (Figure 7A), and the radius of this circle is a variable in the problem. The angle of the vector from the circle's center to the nucleotide 5' to the enclosing base pair (Figure 7A) is also a variable. Base-paired nucleotides along the junction are not constrained to be on the circle, but the straight line connecting the two nucleotides in each base pair must intersect the circle (Figure 7B). These intersections are determined by variables ranging from 0 (coincident with the 5' nucleotide) to 1 (3' nucleotide). The algorithm runs clockwise around the junction constraining nucleotides to lie on the circle, and positioning base pairs based on their intersections. When a move from nucleotide point *p*_1 _to *p*_2 _is performed, we calculate the angle *p*_1_*cp*_2_, where *c *is the circle center. To force a full circle, the sum of these angles is constrained to be 360 degrees. If the multistem junction contains any variable-length regions (which are drawn as arcs), the lengths of these regions are variables in the NLP, allowing some additional flexibility (e.g., Figure 1).

In formulating the objective function, we assume that it is sudden changes in radius that is most visually distracting. For each base-paired nucleotide *n*, we calculate the distance *d*_n _between *n *and *c *(circle center), and the distance *d*_a _between *c *and the nucleotide adjacent to *n *(Figure 7C). The objective function is the sum of squares of the differences *d*_n_-*d*_a_, for all *n*.

The second and third formulations of multistem junction NLPs are similar to one another. In both, the radius of the circle is again a variable, but no points are explicitly constrained to lie on it (Figure 7D). Variables are the *x *and *y *coordinates of the 5' nucleotides of each stem on the junction. However, when two base-paired nucleotides are consecutive (i.e., no unpaired nucleotides are between them), only one variable is used: the angle of the vector from one base-paired nucleotide to the adjacent one. For each junction of unpaired nucleotides between two stems, these nucleotides are drawn along a circle, but each junction's circle has its own independent center and radius (Figure 7E). The center and radius values of such circles are expressed as a constraint in terms of the positions of the base-paired nucleotides flanking the given junction and the constant distance between consecutive nucleotides (see "Default layout of loops").

The objective function for the second formulation measures the deviations of the junctions to the overall multistem junction circle. To approximate the integral, evenly spaced points along each junction are used. The number of points used is *N*+2+3*V *where *N *is the number of unpaired nucleotides, the number 2 reflects the two flanking paired nucleotides and *V *is the number of variable-length regions. The objective function is the sum of the squared differences *d*_pc_-*r*, where *d*_pc _is the distance between a junction point *p *and the main circle's center *c*, and *r *is the radius of the main circle (Figure 7E). The third formulation's objective function uses the idea that it is deviations in slope that are most jarring. Thus, it measures the deviations at regularly spaced points between the vector from the point to the main circle's center and the vector from the same point to the junction's circle center. The objective function is the sum of squared differences between the cosine of the angle between the two vectors and 1 (which represents equal angles).

Nucleotides can be forced to align horizontally by constraining their *x *coordinates to be equal. Using scalar projections, R2R allows alignment at arbitrary angles (not just horizontal), and also allows aligning the centroids of multiple nucleotides (e.g., Figure 3). These features apply to all NLP formulations.

## Conclusions

R2R has sufficient functionality to draw a wide variety of RNA structures and greatly reduce the time necessary to create aesthetic and readable diagrams, which will become increasingly important as more noncoding RNAs are discovered.

## Availability and requirements

• **Project name: **R2R

• **Project home page: **http://breaker.research.yale.edu/R2R

• **Operating system(s): **Platform independent. Note: R2R is only tested using the GNU C++ Compiler.

• **Programming language: **C/C++ and Perl.

• **Other requirements: **CFSQP is needed for some methods of automated layout of multistem junctions. Other aspects of R2R will function with or without CFSQP. CFSQP is free for research and development purposes.

• **License: **GNU General Public License.

• **Any restrictions to use by non-academics: **None.

## Authors' contributions

ZW created the software and drew consensus diagrams based on design principles initially articulated by RRB. ZW and RRB wrote the manuscript, and both authors read and approved its final version.

## Supplementary Material

Additional file 1 **Source code and example input and output files**. C++ and Perl source code is provided. Installation and usage instructions are given in the user manual (Additional file 2), which is also a part of this archive. Example input files for R2R are included in the "demo" subdirectory of this archive, and R2R's raw output on these examples in PDF and SVG format is supplied in the "demo/output-pdf" and "demo/output-svg" subdirectories. Files can be retrieved from the tgz archive file using programs such as WinZip (Windows), StuffIt Expander (Mac) or the tar/gzip commands (UNIX). Click here for file

Additional file 2 **R2R user manual**. User manual explaining R2R installation and usage (also available within Additional file 1). Click here for file

Additional file 3 **Generic annotations for use in drawings, PDF format**. This file contains a generic legend for R2R drawings and some annotations we frequently use, in PDF format. It can be imported into Adobe Illustrator or CorelDRAW. Click here for file

Additional file 4 **Generic annotations for use in drawings, SVG format**. The same content as Additional file 3, but in SVG format. Suitable for import into Inkscape. Click here for file
