# Supplementary material for: R2R - software to speed the depiction of aesthetic consensus RNA secondary structures
Source: BMC Bioinformatics. 2011 Jan 4;12:3. doi: 10.1186/1471-2105-12-3 (PMC3023696; doi:10.1186/1471-2105-12-3)

Generic legend

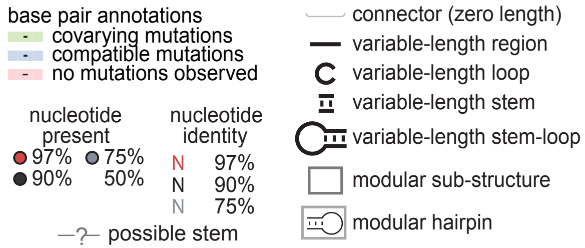

R = A or G. Y = C or U. “nt” = nucleotides. “P1” = pairing element 1. “SD” = Shine-Dalgarno (predicted ribosome-binding site). “start” = start codon.

Example with pseudoknot and modular structure

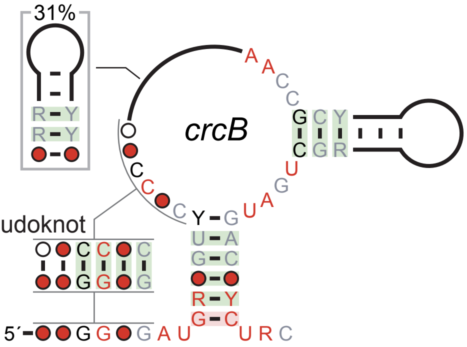

Putting “pseudoknot” over the connector

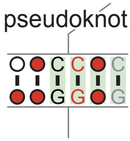

Example with labels for stem, SD & start

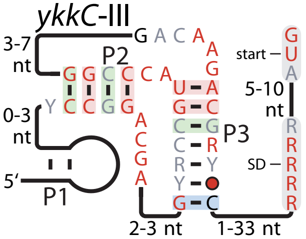

Supplement: Additional file 3 — Generic annotations for use in drawings, PDF format. This file contains a generic legend for R2R drawings and some annotations we frequently use, in PDF format. It can be imported into Adobe Illustrator or CorelDRAW. [file 1471-2105-12-3-S3.PDF]
